# Importance of the C-terminal histidine residues of *Helicobacter pylori* GroES for Toll-like receptor 4 binding and interleukin-8 cytokine production

**DOI:** 10.1038/srep37367

**Published:** 2016-11-21

**Authors:** Haur Lee, Yu-Lin Su, Bo-Shih Huang, Feng-Tse Hsieh, Ya-Hui Chang, Shiou-Ru Tzeng, Chun-Hua Hsu, Po-Tsang Huang, Kuo-Long Lou, Yeng-Tseng Wang, Lu-Ping Chow

**Affiliations:** 1 Graduate Institute of Biochemistry and Molecular Biology, College of Medicine, National Taiwan University, Taipei, 100, Taiwan; 2 Department of Agricultural Chemistry, National Taiwan University, Taipei, 106, Taiwan; 3 Graduate Institute of Oral Biology, College of Medicine, National Taiwan University, Taipei, 100, Taiwan; 4 Department of Biochemistry, Kaohsiung Medical University, Kaohsiung, Taiwan

## Abstract

*Helicobacter pylori* infection is associated with the development of gastric and duodenal ulcers as well as gastric cancer. GroES of *H. pylori (Hp*GroES) was previously identified as a gastric cancer-associated virulence factor. Our group showed that *Hp*GroES induces interleukin-8 (IL-8) cytokine release via a Toll-like receptor 4 (TLR4)-dependent mechanism and domain B of the protein is crucial for interactions with TLR4. In the present study, we investigated the importance of the histidine residues in domain B. To this end, a series of point mutants were expressed in *Escherichia coli*, and the corresponding proteins purified. Interestingly, H96, H104 and H115 were not essential, whereas H100, H102, H108, H113 and H118 were crucial for IL-8 production and TLR4 interactions in KATO-III cells. These residues were involved in nickel binding. Four of five residues, H102, H108, H113 and H118 induced certain conformation changes in extended domain B structure, which is essential for interactions with TLR4 and consequent IL-8 production. We conclude that interactions of nickel ions with histidine residues in domain B help to maintain the conformation of the C-terminal region to conserve the integrity of the *Hp*GroES structure and modulate IL-8 release.

*Helicobacter pylori (H. pylori)* is a Gram-negative, microaerophilic spiral bacterium that infects nearly half of the global population^1^,^2^. Colonization of *H. pylori* in the human gastroduodenal mucosa leads to chronic gastritis and peptic ulcer, and is an important risk factor for gastric cancer^3^,^4^,^5^. The World Health Organization (WHO) classified *H. pylori* as a class I carcinogen based on the strong association between the bacterial infection and gastric cancer^6^,^7^,^8^,^9^. The most remarkable feature of persistent *H. pylori* infection is its induction of inflammatory responses, an important risk factor for malignancy^9^. The inflammatory cytokines triggered by *H. pylori* infection include TNF-α, IL-1β, IL-6 and IL-8 secreted by gastric epithelial and immune cells through activation of transcription factor NF-κB^10^,^11^,^12^,^13^,^14^,^15^.

Several reports have demonstrated increased IL-8 response to *H. pylori*, both *in vitro*^16^ and *in vivo*^15^. IL-8 is a key chemokine that acts as a major regulator of inflammatory cell recruitment^17^. Gastric mucosal IL-8 levels are positively correlated with degree of stomach corpus inflammation^17^, and IL-8 is also highly increased in gastric cancer^18^. IL-8 is the most highly upregulated inflammatory gene by gastric epithelial cells in response to *H. pylori* infection. A number of virulence factors of *H. pylori*-induced host IL-8 production have been identified, including cytotoxin-associated gene A (cagA)^19^, outer inflammatory protein A (OipA)^20^, adherence-associated lipoproteins A and B (AlpA, B)^21^, BabA^22^ and heat-shock protein 60 (HSP60)^23^. Additionally, we previously reported that *H. pylori* GroES (*Hp*GroES) stimulates the production of several proinflammatory cytokines in human peripheral blood mononuclear cells (PBMC) and triggers IL-8 production in gastric epithelial cells^24^. *Hp*GroES was also identified as a dominant gastric cancer-related antigen with significantly higher seropositivity in gastric cancer patients, compared with gastritis and duodenal ulcer patients^24^. The recently characterized, mechanism of *Hp*GroES-induced IL-8 production by gastric carcinoma cells involves activation of the MAPK and NF-κB pathways via a TLR4-dependent mechanism^25^.

GroES, also known as heat shock protein (HSP) A, is an essential protein in *H. pylori*. It is a member of the co-chaperonin family of the GroEL/GroES complex which assist in the function of protein folding^26^,^27^. *Hp*GroES has a highly conserved region (domain A) and a unique C-terminal extension (domain B), which is absent from all other GroES-like proteins, except the closely related *H. acinonychis*^27^,^28^. Domain B of *Hp*GroES contains 4 Cys residues rarely presented amino-acid in the GroES protein family that form disulfide bonds at C94–C111 and C95–C112 to generate a lasso-like structure^29^. Additionally, domain B contains a histidine-rich motif with 8 histidine residues, which bind two nickel ions per GroES monomer. This metal binding ability is associated with nickel homeostasis and protection of cells from higher concentrations of nickel ion^27^,^28^,^30^. In addition, *Hp*GroES binds TLR4 on the cell membrane only in the presence of domain B. *Hp*GroES lacking the disulfide bonds of domain B is unable to interact with TLR4 and induce IL-8 production^25^. Mutational analyses of the Cys residues in domain B revealed that these residues are crucial for interaction with the TLR4 receptor. Thus, conformational changes appear important for domain B binding to TLR4 and induction of IL-8 release.

We previously demonstrated that domain B of *Hp*GroES contributes to GroES-induced IL-8 secretion through a TLR4-dependent signaling pathway^25^. It was shown that a synthetic peptide corresponding to the cysteine /histidine rich sequence of domain B *Hp*GroES has high affinity to nickel ions^31^. To further ascertain the significance of the eight histidine residues of *Hp*GroES domain B in TLR4 binding, metal binding, and IL-8 cytokine production, we conducted site-directed mutagenesis studies and characterized the mutant proteins for bioactivity, TLR4 receptor binding ability, and conformational changes. H100, H102, H108, H113 and H118 of domain B were identified as the key residues affecting IL-8 production and TLR4 binding. These histidine residues were capable of binding nickel ion and four of five residues are required for maintaining local structural integrity. Finally, SAXS shape predictions demonstrated that *Hp*GroES assembles into a ring-shaped homoheptamer with an internal tunnel. Within this ring, these residues maintain the domain B orientation of the *Hp*GroES structure, in turn, ensuring the correct conformation of the C-terminal region for interactions with TLR4.

## Results

### Purification and characterization of WT and histidine mutants of HpGroES

**Figure 1:**
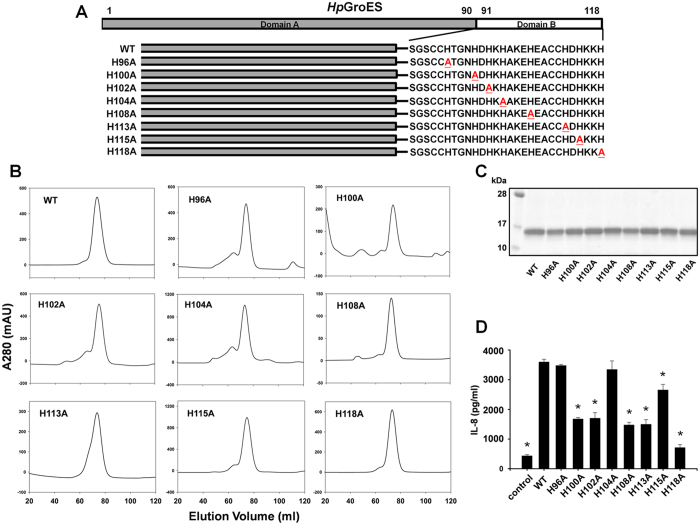
Purification and functional characterization of WT and histidine mutant proteins. **(A)** Schematic representation of the domain structures and domain B sequences (residues 91-118) of WT and histidine mutants. Mutation sites of histidine to alanine are underlined and marked in red. **(B)** FPLC profiles of WT and histidine mutant proteins on a size exclusion column, HiLoad 16/600 Superdex 200 pg. A_280_ was monitored to determine protein elution and purity. **(C)** Purified His-tagged fusion WT and histidine mutants were separated via 15% SDS-PAGE and stained with Coomassie Blue. A 1 μg protein sample was loaded from each elution fraction. **(D)** KATO-III cells were incubated for 16 h with 0.05 μM WT and histidine mutants, and IL-8 levels in the culture supernatant measured using the Quantikine ELISA assay kit. Data are presented as means ± SD of three experiments. *p < 0.05, compared to WT.

*Hp*GroES domain B is essential for IL-8 release by gastric epithelium cells^24^. To examine whether the histidine residues of domain B participate in IL-8 release, we performed site-directed mutagenesis of each of the eight histidines within domain B of *Hp*GroES. The WT and mutant sequences are presented in Fig. 1A. WT and mutants of *Hp*GroES were purified using a Ni-chelating column, followed by size-exclusion chromatography, yielding an oligomeric form (major peak) and a small shoulder peak corresponding to a unstable higher order form (Fig. 1B). Each major peak of the elution volume profile is consistent with a heptameric form which is stable and monodispersed at 4 °C for 2 or 3 days. The yield of purified WT and mutants of *Hp*GroES from per liter of *E. coli* cell was about 10–20 mg protein. SDS-PAGE of purified monomeric WT and mutant proteins revealed a major band with a molecular mass of ~17 kDa (Fig. 1C). The identities of purified WT and histidine mutants were further confirmed using mass spectrometry. To determine whether the histidine mutants activate IL-8, KATO-III cells were incubated with 0.05 μM WT or histidine mutant protein, and cell culture supernatants collected for measurement of IL-8 levels. H96A, H104A and H115A mutants exhibited IL-8 production ability that was not significantly different from wild type, whereas H100A, H102A, H108A, H113A, and H118A mutants displayed only 20–50% IL-8 production relative to WT protein (Fig. 1D). Our results indicate that H100, H102, H108, H113, and H118 residues of *Hp*GroES are crucial for IL-8 release from KATO-III cells.

### WT and histidine mutants bind to TLR4 on KATO-III cell membranes

**Figure 2:**
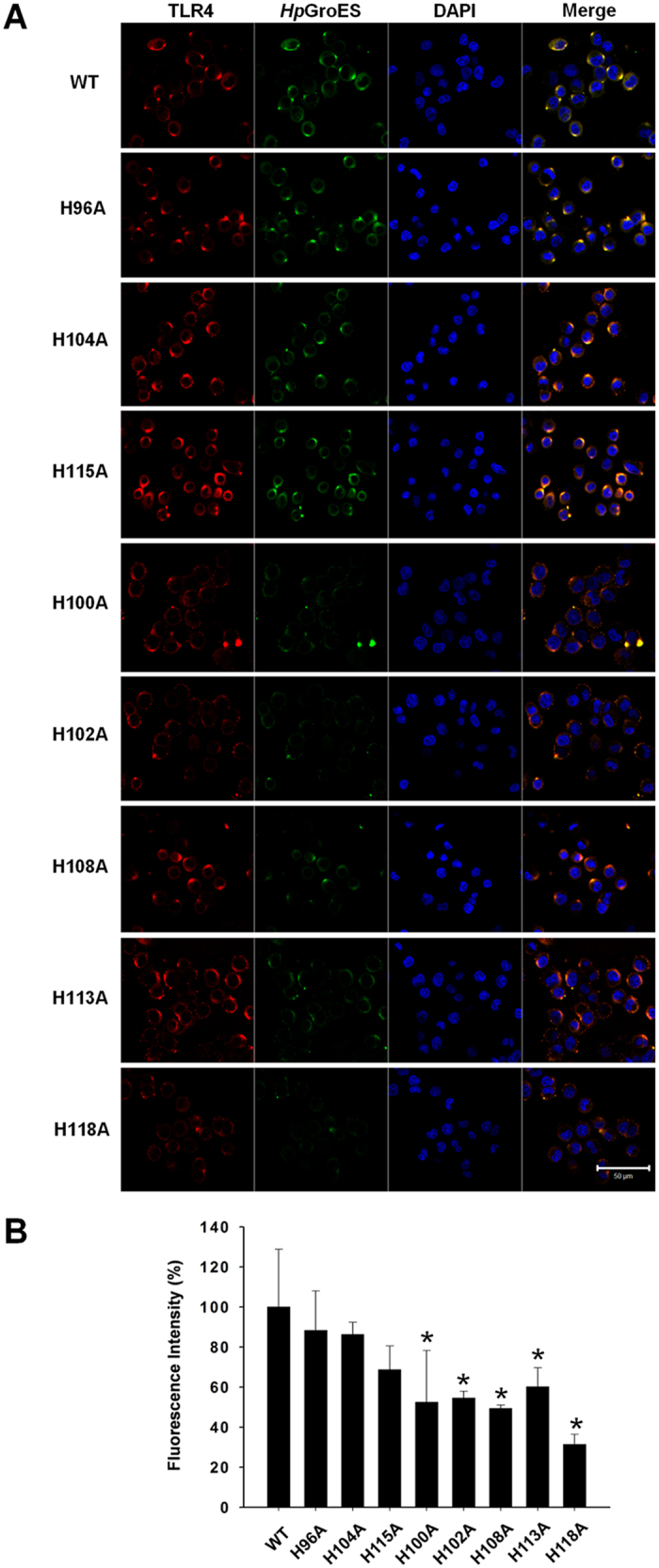
Binding abilities of WT and histidine mutant proteins to TLR4 on KATO-III cell membranes evaluated via confocal microscopy. **(A)** KATO-III cells were incubated for 1 h with 0.05 μM WT or histidine mutants, fixed and double-stained with anti-TLR4 (red) and anti-*Hp*GroES antibody (green). DNA was counterstained with DAPI (blue). The merged images are presented in the right panel, and the quantified percentage of colocalization of WT and histidine mutants and TLR4 in **(B).** Data are representative of three independent experiments. *p < 0.05 compared to WT.

Previous studies have demonstrated that *Hp*GroES binds TLR4 on the cell surface^25^. To further establish the binding abilities of WT and mutant proteins to TLR4 on the cell surface, KATO-III cells were incubated with 0.05 μM WT or histidine mutants, and the localization of *Hp*GroES and TLR4 determined via confocal microscopy. As shown in Fig. 2A, WT, H96A, H104A, and H115A mutants significantly colocalized with TLR4 on the cell surface, whereas H100A, H102A, H108A, H113A, and H118A mutants displayed only partial colocalization with TLR4. Quantification of colocalization of mutant proteins with TLR4 disclosed a 50 to 80% decrease, compared with WT (Fig. 2B). Based on the data, we propose that H100, H102, H108, H113, and H118 residues of *Hp*GroES participate in TLR4 binding.

### Binding affinities of WT or histidine mutants to TLR4

**Figure 3:**
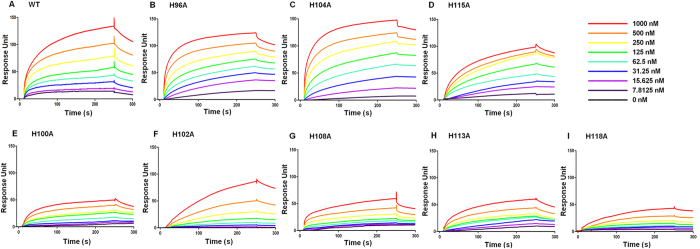
Surface plasmon resonance (SPR) assay of specific binding affinities of WT and histidine mutants to immobilized TLR4. After immobilization of TLR4 on the CM5 sensor chip surface, WT and histidine mutant samples were injected over immobilized TLR4 at a flow rate of 30 μl/min at 25 °C. PBS with 0.05% P20 running buffer was injected, and the response in resonance units recorded for 240 s as a function of time. Protein samples were replaced in running buffer for 480 s for disassociation of bound *Hp*GroES. SPR sensograms were obtained after injection of serial dilutions of WT **(A)** and histidine mutants **(B–I)** (concentration: 0–1000 nM, top right) in running buffer. Data are representative of three independent experiments. The *K*_d_ and *B*_max_ values of protein samples are shown in Table 2.

Surface plasmon resonance (SPR) analysis was performed to examine the direct interactions between WT or histidine mutants and TLR4. TLR4 was coated on the sensor chip and probed with WT or histidine mutant proteins. As shown in Fig. 3A, WT protein bound to TLR4 in a dose-dependent manner. To further identify the critical residues of *Hp*GroES required for interactions with TLR4, we examined the abilities of the mutant proteins to interact with TLR4. Notably, H96A, H104A and H115A proteins were capable of binding TLR4 in a dose-dependent manner (Fig. 3B–D), whereas binding of H100A, H102A, H108A, H113A and H118A mutants to TLR4 was significantly affected (Fig. 3E–I). Saturation curves were further generated (Supplementary Figure. S1); the *K*_d_ values of WT, H96A, H104A and H115A proteins for binding to TLR4 were calculated as 0.10, 0.11, 0.10 and 0.15 μM, respectively (Table 1). In contrast, H100A, H102A, H108A, H113A, and H118A mutant proteins failed to bind TLR4 (Table 1). These results provide direct evidence that mutation of histidine residues at positions 100, 102, 108, 113 and 118 induces significant effects on receptor binding, further leading to reduced IL-8 production.

### Binding of nickel to WT or histidine mutant proteins

**Figure 4:**
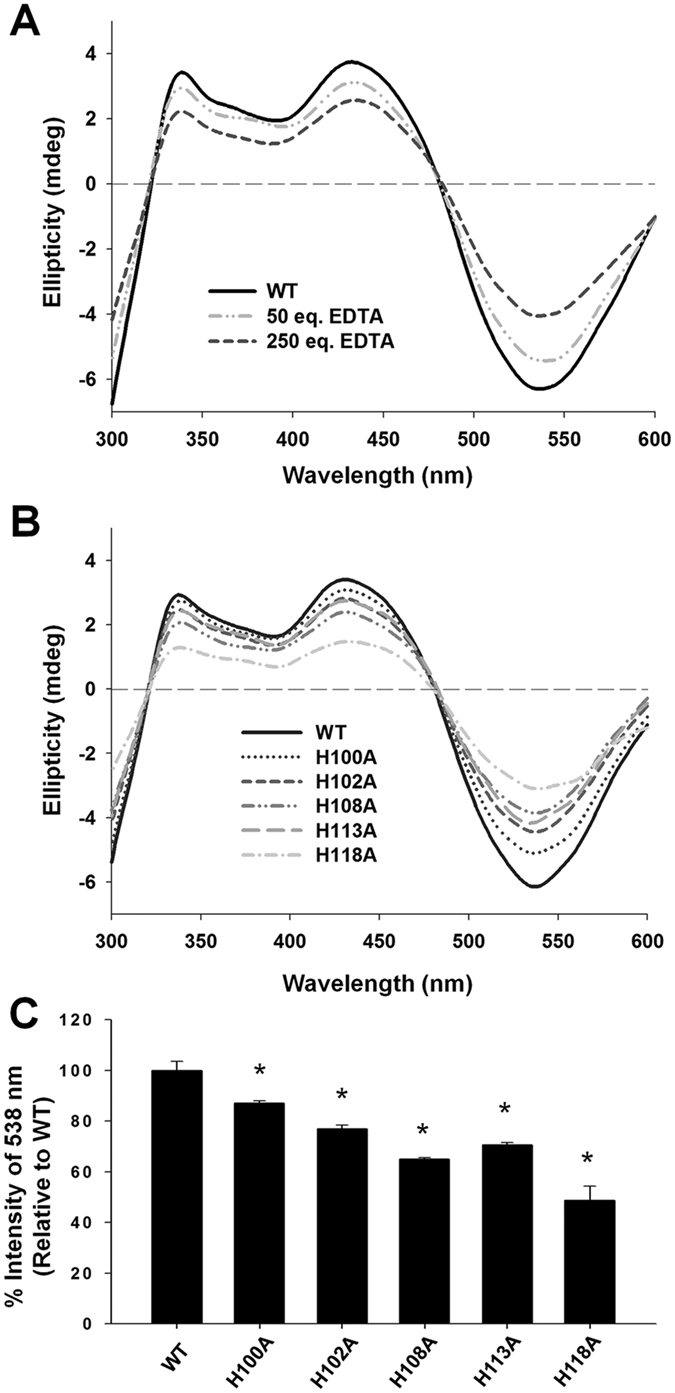
Visible Circular dichroism (CD) spectra of Ni^2+^ bound to WT and histidine mutant proteins. **(A)** Visible CD spectra (300–600 nm) of Ni^2+^ bound to 20 μM WT in 150 mM NaCl, 25 mM potassium phosphate,pH 7.4, 1 mM DTT and 0.3% glycerol with 50 and 250 molar equivalents of EDTA. The differences in ellipticity were approximated at 440 and 538 nm. **(B)** Visible CD spectra of Ni^2+^ bound to 20 μM WT and histidine mutants. The differences in ellipticity values were approximated at 440 and 538 nm, indicating different amounts of Ni^2+^ binding to the proteins. Data are representative of three independent experiments. **(C**) Intensities of the negative band at 538 nm of WT and histidine mutants. Data were normalized and compared to WT (whereby intensity of WT was defined as 100%).

To identify the histidine residues that participate in nickel ion binding and further influence TLR4 interactions and IL-8 production, we assessed the nickel ion binding abilities of histidine mutants via CD spectroscopy, measuring ellipticity at 440 nm and 538 nm that represented the d-d transition when formation of nickel-protein complex^31^,^32^,^33^,^34^,^35^. WT protein reaction with the metal chelating agent, EDTA, was used as the reference. After the addition of 50 and 250 molar equivalents of EDTA, the CD signals at 440 nm and 538 nm of WT were sequentially altered (Fig. 4A). The H96A, H104A and H115A mutants displayed no spectral differences compared to WT, signifying that these mutations do not affect binding ability of the protein to nickel ion (data not shown). However, a subset of mutants displayed changes in intensity at 440 nm and 538 nm in the following order: H100A, H102A, H113A, H108A and H118 A (Fig. 4B). Quantification of the percentage of nickel ion binding ability revealed a slight reduction in the binding ability of H100A (~82% of WT) but significant reduction for H102A, H108A, H113A, and H118A mutants (by 30–50%), compared with WT (Fig. 4C). Thus, these five histidine residues appear critical for nickel binding by *Hp*GroES.

### Differences in secondary structure between WT and histidine mutant proteins

**Figure 5:**
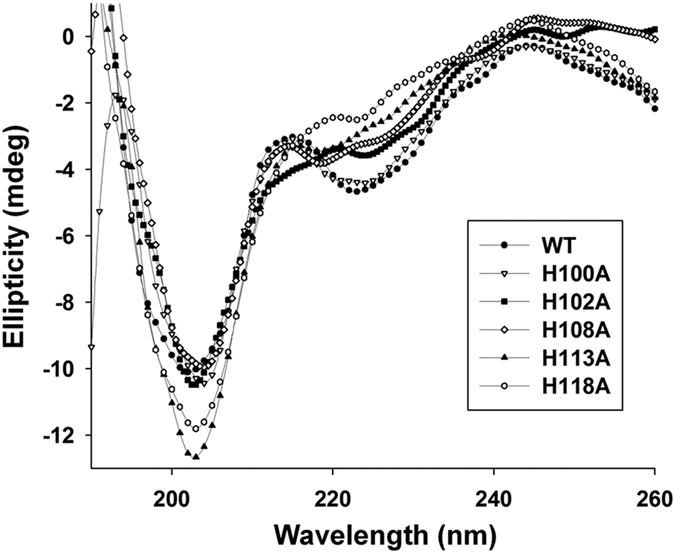
Far-ultra violet (UV) circular dichroism (CD) analysis of the differences between histidine mutant and WT proteins. Far-UV CD spectra (190–260 nm) of H100A, H102A, H108A, H113A, H118A and WT proteins were obtained in 25 mM sodium phosphate buffer, pH 7.4, with 1 mm path length of the quartz cuvette at 4 °C. The data collection bandwidth was set to 1 nm. The changes in ellipticity values at 200–210 nm and 220–225 nm range were monitored indicated different secondary structures of histidine mutants and WT in solution. Further secondary structure variations in percentages for each histidine mutant and WT determined using the CDSSTR program are presented in Supplementary Table SII. Data are representative of three independent experiments were subjected to smoothing.

Data from vis-CD and SPR analyses showed that the H100A has a slight reduction in the nickel binding ability, but lacks the ability to bind TLR4. Additionally, H102A, H108A, H113A and H118A mutants lost the ability to bind nickel and leading to reduced binding to TLR4. To ascertain whether these mutations induced structural changes, we compared the secondary structures of WT and histidine mutants using far-UV CD. As shown in Fig. 5, far-UV CD spectra showed two minima peaks of the WT protein at 200–210 nm and 220–225 nm range. The far-UV CD spectrum of the H96A, H104A and H115A were identical to that of WT (data not shown). In addition, the far-UV CD spectrum of the H100A was similar to that of WT, implying no significant differences between the two structures. However, the far-UV CD spectra of H102A, H108A, H113A and H118A mutant proteins demonstrated changed intensity of the two negative bands, indicating different secondary structures of these histidine mutants and WT in solution, as shown in Supplementary Table SII. Based on these observations, we propose that H102, H108, H113 and H118 residues bind to nickel ion and participate in stabilizing the conformation at domain B of *Hp*GroES.

### Solution structural analysis of WT and histidine mutant proteins with SAXS modeling

**Figure 6:**
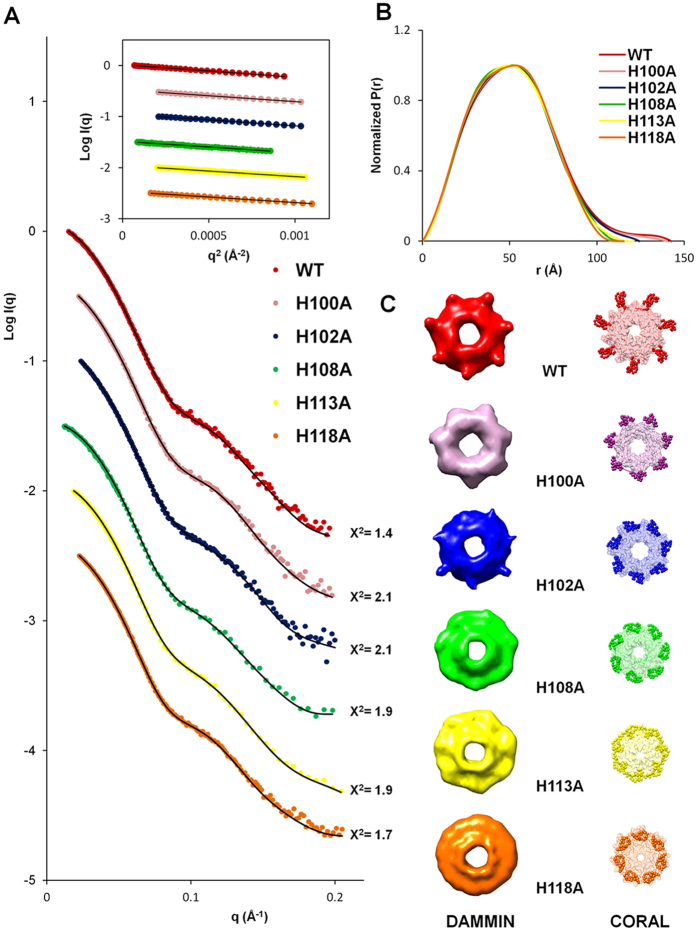
SAXS data analysis and *ab initio* models of WT and variant proteins. **(A)** SAXS intensity profiles showing the log of X-ray scattering intensity (logI) as a function of the scattering vector q for experimental scattering data. The Guinier curve-fitted lines for Rg values to the experimental data are superimposed (Inset). Both curve profiles were corrected and offset for clarity of presentation, and showed good quality of protein samples. **(B)** Overlay of normalized p(r) distribution plot calculated using Primus for WT and variants. **(C)** The *ab initio* models of averaged molecular envelopes from DAMMIN (left) and rigid body models from CORAL (right) are shown in the same colors as those in panel A. The models showed good fit with the experimental SAXS curve in **(A)** (χ^2^ = 1.4–2.1).

Next, we employed SAXS to compare the molecular shape and particle dimensions of WT and above mutant proteins. Normalized scattering curves for each protein are shown in Fig. 6A. The linearity in the low q Guinier region in Guinier plot indicated that protein did not undergo aggregation (Fig. 6A inset). Estimates of Rg and *D*_max_ values were obtained from Guinier plot and P(r) curves (Fig. 6A inset and B). The results showed Rg and *D*_max_ values are summarized in Table 2. H96A, H104A and H115A mutant proteins exhibits very similar Rg and *D*_max_ values to the WT protein. H100A mutant proteins also displayed similarity Rg values and *D*_max_ values to the WT protein. However, smaller Rg Guinier (39.43 Å, 38.52 Å, 38.61 Å, 38.27 Å) and *D*_max_ values (124.4 Å, 117.4 Å, 121.1 Å, 115.4 Å) were obtained for H102A, H108A, H113A, and H118A mutant proteins, respectively. Our data indicate that WT, H96A, H100A, H104A and H115A share an extended structure while H102A, H108A, H113A, and H118A have compact structures. Envelope models were further constructed from the experimental scattering profiles using DAMMIN and the domain B structure generated with 28 dummy residues in the C-terminus with CORAL model (Fig. 6C). The models indicate that both WT and H100A proteins contain seven major extruded structures located within domain B. However, in the H102A, H108A and H113A mutants, the domain B extrusions were significantly shorter and formed an oblate shape. In the case of H118A, the seven extruded domain B structures completely disappeared, leading to a compact oblate shape.

### Determination of nickel coordination by molecular modeling and mutagenesis

Finally, the nickel coordination geometry was carried out by molecular modeling and mutagenesis analysis. Model of domain B was generated as described in Supplementary Method. The binding mode of two nickel ions coordination geometries within domain B of *Hp*GroES was shown in Supplementary Fig. S2. The results indicated that three residues (H100, H102 and H108) interacted with one nickel ion, and the two residues (H113 and H118) interacted with another nickel ion. To further address the possible of nickel coordination, the double mutants H100A/H102A, H100A/H108A, H102A/H108A, H113A/H118A were constructed by site-directed mutagenesis and different mutant proteins were purified to homogeneity. As expected, a subset of double mutants H100A/H102A, H100A/H108A, H102A/H108A, H113A/H118A exhibited greatly reduced affinity for nickel ion (by 40–50%) compared with WT (Supplementary Fig. S3). The data indicated that the nickel prefer coordination motif as observed from the structure was HDH(X)nH, wherein the underlined sites represent the nickel coordinating residues. The results provide the possibility of using nickel coordination geometry to enhance the structure stability of domain B.

## Discussion

Chronic *H. pylori* infection causes gastric cancer via the expression of virulence factors and induction of chronic inflammatory responses that result in increased levels of proinflammatory cytokines^9^,^10^,^11^,^12^. Hsp (heat shock proteins) are conserved protein families expressed in response to environmental stress. These proteins are involved in chaperone functions in assisting translocation and facilitating proper protein folding^26^,^36^,^37^. Several bacterial Hsps have been shown to induce proinflammatory cytokines^38^,^39^,^40^,^41^,^42^,^43^,^44^. The Hsp60 family shares extensive structural homology and enhances cytokine production from various bacterial types^45^. In the majority of bacteria, the GroES family represents small-sized Hsps which inhibit binding to receptors^46^. *Hp*GroES is specifically able to induce IL-8 secretion^24^ and possesses a unique domain B at the C-terminus that is absent from other known GroES homologs^27^. IL-8 is an important chemotactic and activating factor for neutrophils^47^. The cytokine plays a role in gastric mucosal injury caused by *H. pylori* and is correlated with the development of chronic gastritis^16^. Domain B of *Hp*GroES contributes to *Hp*GroES-induced IL-8 secretion through a TLR4-dependent mechanism^25^. Domain B displays a unique conformational loop structure generated by two disulfide bonds referred to as the four-cysteine ring (4CR) motif. The two consecutive disulfide bridges form a rigid scaffold that dramatically affects the topology of the loop structure of domain B^29^. The 4CR motif forms a short loop generating a conformation optimal for receptor recognition^25^. Upon disruption of the two disulfide bonds, *Hp*GroES domain B loses its conformation, leading to loss of binding to TLR4 and consequent IL-8 production^25^. Based on these findings, it is proposed that the conformation of domain B of *Hp*GroES is crucial for TLR4-mediated IL-8 induction.

The role of disulfide bonds formed by the four cysteine residues of *Hp*GroES domain B in TLR4 binding has been explored previously^25^. In the current study, we investigated the importance of histidine residues of domain B in *Hp*GroES binding to TLR4 and consequent IL-8 production via site-directed mutagenesis of the eight histidine residues within this region. Our results demonstrated that H96A, H104A, and H115A mutants retain similar IL-8 secretion activities to wild type *Hp*GroES. The *K*_d_ values of the H96A, H104A, and H115A mutants for TLR4 were determined as 0.11 μM, 0.10 μM and 0.15 μM, respectively, suggesting that these residues are not required for TLR4 binding. The far-UV CD spectra for WT and three mutant proteins were essentially identical, indicating that these substitutions do not affect the spatial arrangement of the peptide backbone. In contrast, H102A, H108A, H113A, and H118A mutants induced a marked reduction in IL-8 production, lost their ability to bind TLR4, and displayed alterations in secondary structure. Notably, however, mutation of H100 had no effect on the secondary structure of the protein, although binding of TLR4 was weaker and IL-8 production decreased. These results suggest that specific tertiary structures involving H100, H102, H108, H113 and H118 are important for binding to TLR4.

*Hp*GroES functions as a specialized nickel chaperone through domain B^48^,^49^. This nickel binding ability is important for nickel storage and detoxification^27^,^28^,^30^,^50^. Deletion of domain B of *Hp*GroES leads to decreased intracellular nickel content and increased nickel sensitivity^28^. The lasso-like structure of domain B of *Hp*GroES provides a suitable conformation for binding to nickel ions^29^. To further clarify size and shape of domain B of *Hp*GroES, a SAXS experiment was performed. DAMMIN and CORAL model analyses revealed that *Hp*GroES forms a circular heptameric ring structure similar to GroES of *E. coli*^51^,^52^. We observed seven major extended radial symmetrical structures extending from the core domain that were absent in the GroES structures of other species^53^,^54^,^55^,^56^ and corresponded to the C-terminal portion of the *Hp*GroES. The Rg value (34.16 Å) (data not shown) of the domain A of *Hp*GroES without the extended structure was in accordance with that of GroES of *E. coli* (34.0–35.0 Å) reported previously^51^,^52^,^57^. Moreover, long tail distribution of the curve in the P(r) plot supported the extended structure of *HpGroES*. DAMMIN and CORAL models showed that domain B is contained within the extruded C-terminal portion. Thus, the extended structures of these models represent a close approximation of domain B of *Hp*GroES, and nickel ion binding to domain B may be required to maintain its conformation.

As reported previously, the nickel binding motif of domain B of *Hp*GroES, HX_4_DH, is located at positions 96–102 (HTGNHDH) according. Substitution of DH with AA results in decreased intracellular nickel content and reduced nickel tolerance^28^,^58^. In the current study, three histidine mutants (H96A, H104A, and H115A) showed similar binding to nickel ions as WT, while other mutants (H100, H102, H108, H113 and H118) lost 20–50% reductions of nickel binding. Two separate H-D-H-X-H nickel binding motifs identified in domain B. Among the mutants of histidines located within the first motif, 100–108 (HDHKHAKEH), nickel binding ability decreased 20% but conformation of H100A were similar to that of WT. SAXS models revealed seven extended structures as WT. This mutation did not affect the structural conformation of the protein although IL-8 secretion and TLR4 binding abilities were significantly decreased. The inability of H100A to bind TLR4 can therefore be attributed to replacement of the histidine at position 100 with Ala and not structural changes. The positive charge and the length of the side chain seem to be important for the receptor interaction. However, the specific role of the H100 residue remains to be established. In contrast, the H102A led to structural conformation changes along with decreased nickel binding ability. SAXS data disclosed a decreased size of the extended structures of H102A, with smaller Rg and *D*_max_ values. Among histidines from the first and second motif, 113–118 (HDHKKH), mutation of H108 and H113 displayed significant structural changes with decreased nickel binding ability. SAXS data revealed near-complete loss of the extended structures of domain B, suggesting that the C-terminal conformation is maintained through domain B binding to nickel ions. Furthermore, mutation of H118 in second motif led to significant loss of nickel binding ability. Far-UV CD spectra analysis showed that a ~10% increase in flexible coil structure of H118A than WT. H118A also exhibited the smallest size with disappearance of the extruded structure, signifying dramatic conformational changes. H118A completely lost its natural shape and was rearranged into an overall oblate ring shape. Moreover, IL-8 activity was almost abolished in the H118A implying that this residue is directly involved in binding nickel ions and potentially contributes to C-terminal conformation stability.

In the modeled structure, the first nickel ion is coordinated to three histidine residues (H100, H102 and H108) and the second nickel ion is ligated to H113 and H118 in domain B. Furthermore, various double mutations reduced the nickel ion binding strength significantly as compared to the single mutations, indicating that these five histidine residues can coordinate nickel ion. In general, the nickel ion preferentially adopts planar coordination geometry^31^,^32^,^33^,^35^. The nickel ion has coordination numbers by four ligands^34^. It is reported that histidine, aspartate, glutamate and cysteine are the typically residues to coordinate nickel, among which, histidine is the most frequent over other amino acids^59^. Thus, further research to investigate the other potential nickel ligands is required.

In conclusion, the histidine residues H102, H108, H113 and H118 in domain B of *Hp*GroES participate in binding of nickel ion and maintaining conformational integrity. Mutation of these residues results in disruption of the conformation of extended domain B and further reduces secretion of IL-8 through loss of binding ability with TLR4. Our results collectively highlight critical roles for these histidine residues in terms of contribution to the overall structure of *Hp*GroES protein. Furthermore, the unique C-terminal domain B conformation of *Hp*GroES supports its function as a determinant of *Hp*GroES pathogenesis in the *H. pylori-*induced innate immune response.

## Methods

### Reagents and antibodies

The pQE30 plasmid was obtained from Qiagen (Chatsworth, CA), and the Phusion site-directed mutagenesis system from Thermo Scientific (Waltham, MA). The Ni^2+^-chelating Sepharose column and HiLoad 16/600 Superdex 200 pg column were from GE Healthcare (Kowloon, HK). Triton X-114 and protease inhibitor cocktails were purchased from Sigma-Aldrich (St. Louis, MO). The ultrafiltration membrane (30 kDa cut off) was from Millipore (Bedford, MA). Cell culture medium RPMI was purchased from Hyclone (Cambridge, MA), while FBS, penicillin, and streptomycin were from Life Technologies (Gaithersburg, MD). Quantikine ELISA assay kits for human IL-8 and recombinant human TLR4 protein were from R&D Systems (Minneapolis, MN). Rabbit antibodies to *Hp*GroES were produced in our laboratory^24^. The mouse mAbs against human TLR4 (HTA125) were acquired from BioLegend (San Diego, CA). TRITC-conjugated anti-rabbit and FITC-conjugated anti-mouse IgG antibodies were sourced from Millipore (Bedford, MA).

### Cloning, expression and purification of WT and histidine mutants

The *Hp*GroES gene was cloned using the pQE30 expression vector as described previously^24^. The eight histidine point mutants and four histidine double mutants were generated with the Phusion site-directed mutagenesis system. PCR reactions were carried out with primers whose sequences are listed in Supplementary Table SI. Vectors expressing the mutant GroES constructs were transformed into *E. coli* strain M15. To confirm the presence of the desired mutations, DNA sequences were determined. M15 cells containing the expression vector pQE30 carrying WT and histidine mutant gene fragments were grown to *A*_600_ values of 0.5–0.7, induced with 1 mM isopropyl *β*-D-thiogalactoside (IPTG), and harvested after 4 h at 37 °C. Recombinant proteins were purified on a Ni^2+^-chelating sepharose column. To remove endotoxin, soluble recombinant proteins were purified in buffer containing 1% Triton X-114, loaded onto the column and washed with binding buffer containing 0.1% Triton X-114 for elution of target proteins. The crude protein eluate was dialysis against 150 mM NaCl, 25 mM potassium phosphate, pH 7.4, 1 mM DTT, and 0.3% glycerol with 4 molar equivalence of NiSO_4_ and subjected to chromatography on a HiLoad 16/600 Superdex 200 pg column pre-equilibrated with the same buffer without NiSO_4_. The column was run at a flow rate of 2 ml/min. Fractions (1.5 ml) displaying the correct size of *Hp*GroES were collected and concentrated using Ultrafiltration membrane (30 kDa cut off). For cell culture, protein solution was dialyzed against PBS. Protein concentrations were measured using the BCA protein assay kit (Pierce Biotechnology, Rockford, IL). Purified recombinant proteins were separated via SDS-PAGE and visualized with Coomassie blue.

### Cell culture and cytokine ELISA assay

The human gastric carcinoma cell line, KATO-III, was obtained from the Japan Cancer Research Bank (Tokyo, Japan). Cells were cultured in RPMI 1640 with 10% FBS, 100 μg/ml streptomycin and penicillin at 37 °C in 5% CO_2_. Cell cultures were incubated for 16 h with 0.05 μM WT and histidine mutant and then the cell culture supernatant collected following centrifugation at 1500 rpm for 10 min. Levels of IL-8 in the culture supernatants were measured using the Quantikine ELISA assay kit (R&D Systems, Minneapolis, MN). All experiments were performed in triplicate.

### Immunofluorescence staining

KATO-III cells were grown on coverslips overnight, followed by treatment with 0.05 μM WT and histidine mutants for 1 h. After washing with PBS, cells were fixed with 4% formaldehyde in PBS at room temperature for 15 min and blocked with 5% BSA in PBS for 1 h at room temperature. For immunostaining, cells were incubated with rabbit antibodies against *Hp*GroES (1:200) or mouse mAb against TLR4 (1:200), followed by FITC- or TRITC-labeled secondary antibodies (1:500) and nuclei stained with DAPI (1:1000). Digital images were captured with a TCS SP5 confocal microscope (Leica, Wetzlar, Germany).

### Circular dichroism (CD) measurements

CD spectra of proteins were measured at 4 °C in the far UV (190–260 nm) or visible range (300–600 nm) on a J-810 spectropolarimeter (Japan Spectroscopic Ltd., Tokyo, Japan). The bandwidth and step resolution were set to 0.5 or 1 nm. The optical path length of the cuvette was 1 mm. A quartz cuvette was cleaned by soaking in potassium dichromate solution (10% [w/v] potassium dichromate, 10% [v/v] H_2_SO_4_) and rinsed before use. WT and histidine mutants were diluted to suitable concentrations (20 μM) in 150 mM NaCl, 25 mM potassium phosphate buffer, pH 7.4, 1 mM DTT, 0.3% glycerol for vis-CD or 3 μM in 25 mM sodium phosphate buffer, pH 7.4, for far-UV CD. For each sample, three scans were performed to obtain an average spectrum, which was subtracted from the buffer spectrum to provide a baseline correction. The secondary structure percentages of WT and histidine mutants were determined using the CDSSTR software program (shown in Supplementary Table SII).

### Surface Plasmon Resonance (SPR) Spectroscopy

All SPR experiments were carried out on a Biacore T200 (GE Healthcare, Uppsala, Sweden) with active temperature control at 25 °C following the manufacturer’s protocols. The running buffer used as PBS with 0.05% P20. For protein immobilization, TLR4 (20 μg/ml) in running buffer equivalent to 6000 RU was injected onto a CM5 sensor chip at a flow rate of 5 μl/min. Another flow cell without immobilized TLR4 coating was used to evaluate nonspecific binding. WT and histidine mutants were diluted in running buffer (final concentrations, 1000 to 7.8125 nM) and flowed across immobilized TLR4 for 240 s at a flow rate of 30 μl/min (association). The sample was replaced with running buffer, followed by disassociation of bound *Hp*GroES for 480 s (disassociation). The chip surface was regenerated by injecting 1 M NaCl for 30 s and 10 mM Glycine-HCl, pH 3.5, for 10 s. For all samples, blank injection with buffer alone was subtracted from the resulting reaction surface data. Data were analyzed using Biacore T200 evaluation software and fitted using GraphPad Prism software.

### Small angle X-ray scattering (SAXS) data collection and processing

SAXS measurements were collected at beamline 23A1 of the National Synchrotron Radiation Research Center (NSRRC), Taiwan, with an online size-exclusion high pressure liquid chromatographic (SE-HPLC) system. Typically, 50 μL protein sample (20 mg mL^−1^) was injected into the HPLC column. Next, the sample was directed into a quartz capillary for SAXS measurements thermostatted at 298 K. Data were collected at a rate of 1 frame per 30 s using a Pilatus 1M-F area detector. The energy of the X-ray beam was 15 keV (wavelength λ = 0.8266 Å), and the setup adjusted to achieve scattering *q* values of 0.007 Å^−1^ to 0.3 Å^−1^, *q* defined by 4πλ^−1^sinθ with scattering angle 2θ. Data were corrected for electronic noise, and sample transmission, followed by scaling to absolute intensity *I(q*) in units of cm^−1^ via scattering from water at protein sample conditions^60^. Scattering curves for each protein were used for Guinier analyses using the program Primusqt from the ATSAS package. The pair distribution functions P(r) calculated the radius of gyration (Rg) by integrating the function with r^2^ over all values of r and maximum particle dimensions (*D*_max_) by computing with autoGNOM. Rg estimates were also obtained using Guinier analysis. *Ab initio* modeling was performed with the program DAMMIN to obtain 10 to 15 independent dummy bead models that were subsequently averaged in DAMAVER software. Bead models of molecular envelopes for each structure were converted to surfaces with Chimera. Each DAMMIN model showed good fit to the experimental SAXS curve (χ^2^ = 1.4~2.1) and was calculated with normalized spatial discrepancy (NSD) values in the range 0.5–0.7. To construct the conformation of domain B missing from other crystal or solution structures, CORAL models of WT and histidine mutants were also built based on the domain A template of *Hp*GroES from SWISSMODEL with *Mycobacterium tuberculosis* GroES (PDB: 1P3H) assembled with 28 dummy C-terminal residues. Rigid body modeling into SAXS-derived molecular envelopes was manually performed using the program Chimera.

### Statistical analysis

All experiments were performed in triplicate, and data expressed as means ± SD. Student’s t test was used to determine the significance of differences between treated and control samples. For all tests, data were considered significant at *p* < 0.05.

## Additional Information

**How to cite this article**: Lee, H. *et al.* Importance of the C-terminal histidine residues of *Helicobacter pylori* GroES for Toll-like receptor 4 binding and interleukin-8 cytokine production. *Sci. Rep.* **6**, 37367; doi: 10.1038/srep37367 (2016).

**Publisher’s note:** Springer Nature remains neutral with regard to jurisdictional claims in published maps and institutional affiliations.

## Supplementary Material

Supplementary Information

## Figures and Tables

**Table 1:** *K*_d_ and *B*_max_ values of WT and histidine mutant proteins.

Sample	*K*_d_ (*μ*M)	*B*_max_ (RU)
WT	0.10	133.2
H96A	0.11	142.1
H104A	0.10	168.9
H115A	0.15	132.0
H100A	>1	103.0
H102A	>1	324248.0
H108A	>1	51076.0
H113A	>1	37892.0
H118A	>1	32193.0

**Table 2:** SAXS data analysis of WT and histidine mutants.

Sample	Rg Guinier (Å)	Rg P(r) (Å)	*D*_max_ (Å)
WT	40.82	40.88 ± 0.065	142.7
H96A	40.11	40.23 ± 0.023	140.0
H104A	40.70	40.74 ± 0.072	142.0
H115A	40.52	40.56 ± 0.105	140.1
H100A	40.00	40.04 ± 0.098	140.2
H102A	39.43	39.42 ± 0.052	124.4
H108A	38.52	38.48 ± 0.061	117.4
H113A	38.61	38.57 ± 0.061	121.1
H118A	38.27	38.20 ± 0.032	115.4
