# Supplementary material for: Importance of the C-terminal histidine residues of Helicobacter pylori GroES for Toll-like receptor 4 binding and interleukin-8 cytokine production
Source: Sci Rep. 2016 Nov 21;6:37367. doi: 10.1038/srep37367 (PMC5116745; doi:10.1038/srep37367)
Supplement: Supplementary Information [file srep37367-s1.pdf]

Supplementary Information

Importance of the C-terminal histidine residues of *Helicobacter pylori* GroES for Toll-like receptor 4 binding and interleukin-8 cytokine production

Haur Lee[+], Yu-Lin Su[+], Bo-Shih Huang[+], Feng-Tse Hsieh[+], Ya-Hui Chang[+], Shiou-Ru Tzeng[+], Chun-Hua Hsu[§], Po-Tsang Huang[+¶], Kuo-Long Lou[+¶], Yeng-Tseng Wang[&] and Lu-Ping Chow[+]*

[+] Graduate Institute of Biochemistry and Molecular Biology, College of Medicine, National Taiwan University, Taipei 100, Taiwan

[§]Department of Agricultural Chemistry, National Taiwan University, Taipei 106, Taiwan

[¶]Graduate Institute of Oral Biology, College of Medicine, National Taiwan University, Taipei 100, Taiwan

[&]Department of Biochemistry, Kaohsiung Medical University, Kaohsiung, Taiwan

[*] Corresponding author. chowip@ntu.edu.tw

Supplementary Method

Homology modeling of domain B

We used the domain B sequence (SGSCCHTGNHDHKHAKEHEACCHDHKKH) of *Hp*GroES as our target. The predicting model of domain B from I-TASSER [1]and subsequently applied molecular docking simulation method for the complex structure of the two nickel ions/domain B. Next, autodock via docking software [2] was used to dock the two nickel ions into the domain B structure. The best model was then selected for subsequent molecular dynamics (MD) simulations. Initially, the best model was inserted into the tip3p water box. The MD simulations were performed in the canonical ensemble with a simulation temperature of 310 K, unless stated otherwise, by using the Verlet integrator with an integration time step of 0.002 ps and SHAKE constraints [3] of all covalent bonds involving hydrogen atoms. In the electrostatic interactions, atom-based truncation was performed using the PME method [4], and the switch van der Waals function was used with a 2.00 nm cut-off for atom-pair lists. The complex structure was minimized for 10000 conjugate gradient steps, and was then subjected to a 100 ns isothermal, constant volume MD simulation. The MD calculations were carried out with the Amber14 program with Amber FF99SB and additional nickel-bound force fields [5]. Moreover, the final structures from these simulations were used in the solvated interaction energies (SIE) and alanine scanning calculations.

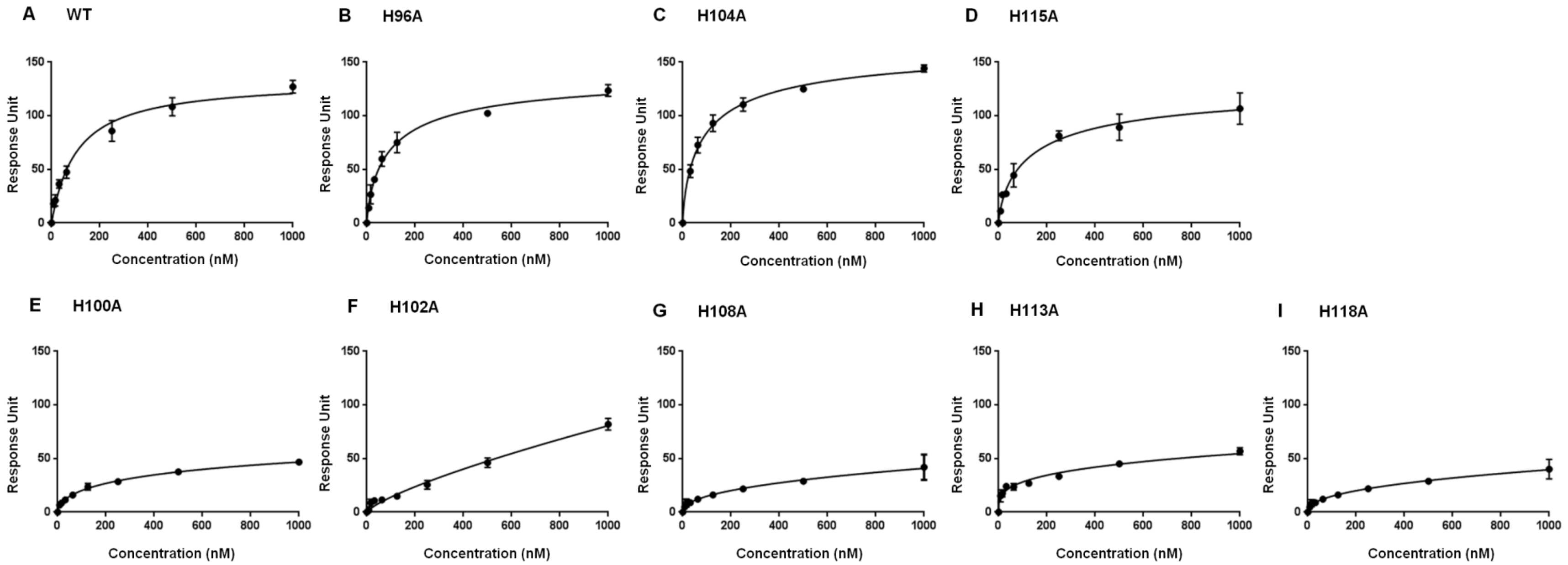

Supplementary Figure S1. The steady state binding affinity analysis of WT and histidine mutants to immobilized TLR4 by surface plasmon resonance (SPR) assay

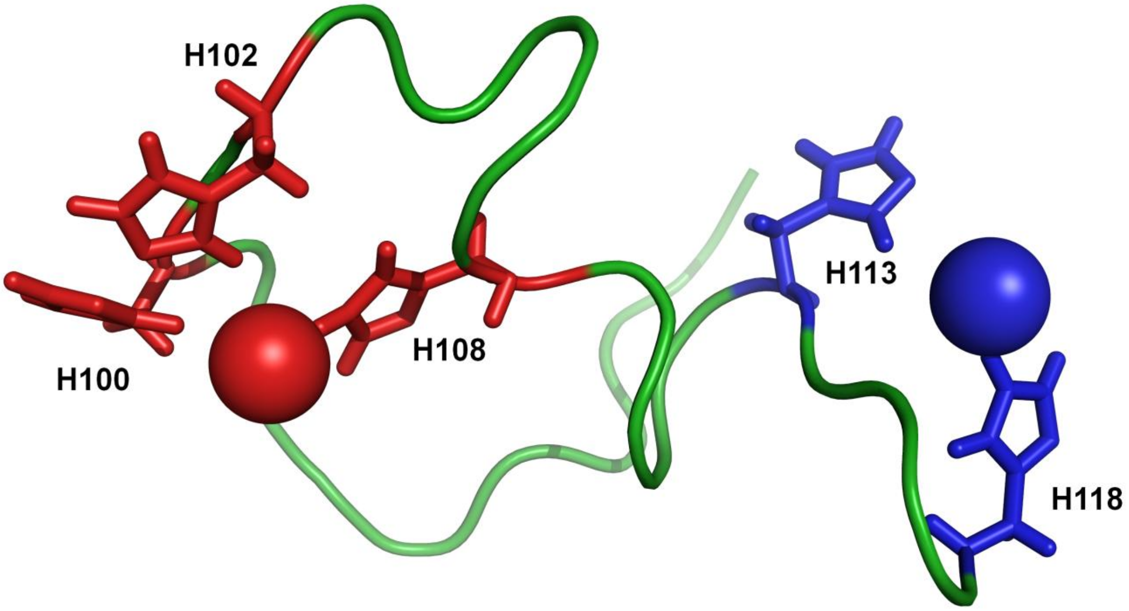

Supplementary Figure S2. Model of the domain B with putative nickel binding residues

The configuration calculated for nickel binding to various histidine residues. The two nickel ions are colored in red and blue.

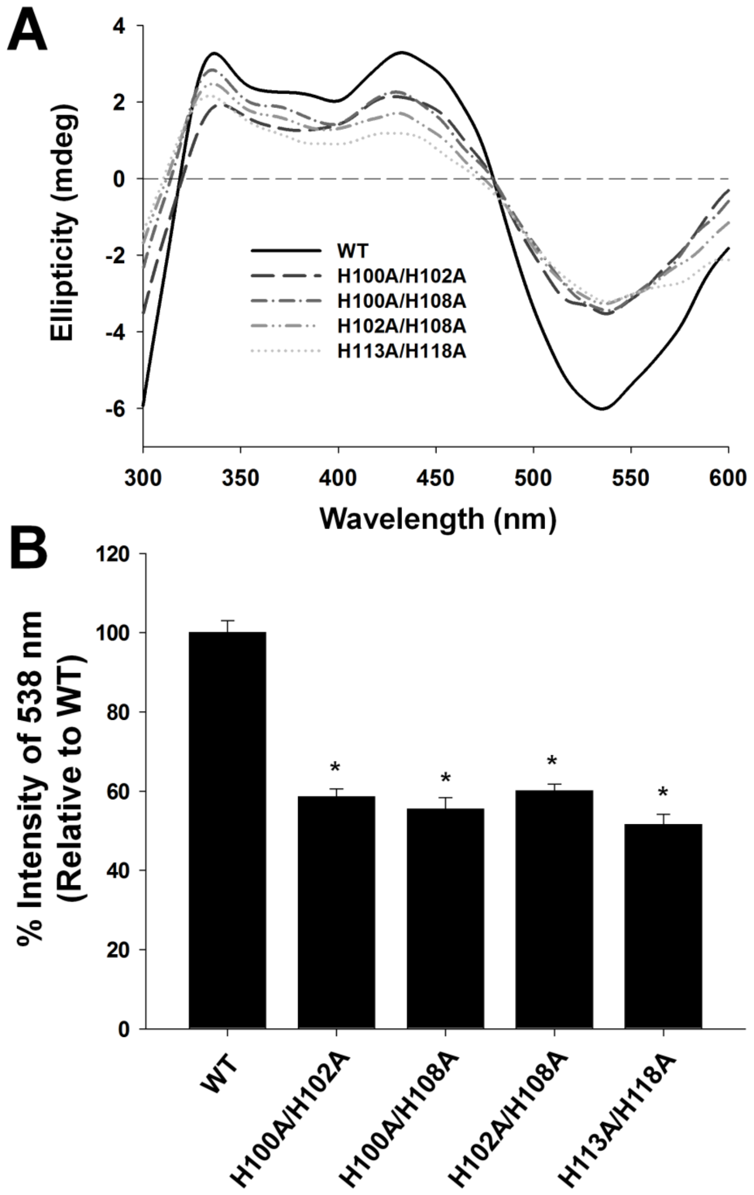

Supplementary Figure S3. Visible Circular dichroism (CD) spectra of Ni^{2+} bound to WT and various histidine double mutants.

(A) Visible CD spectra of Ni^{2+} bound to 20 μM WT and histidine double mutants. The differences in ellipticity values were approximated at 440 and 538 nm, indicating different amounts of Ni^{2+} binding to the proteins. Data are representative of three independent experiments. **(B)** Intensities of the negative band at 538 nm of WT and histidine double mutants. Data were normalized and compared to WT (whereby intensity of WT was defined as 100%).

Supplementary Table SI. Primer sequences used for *Hp*GroES constructs

Construct	Primer Sequence
***Hp*GroES WT**	F: 5-GGATCCATGAAGTTTCAGCCATTAGGAGA-3
	R: 5-GGTACCTTAGTGTTTTTTGTGATCATGACA-3
***Hp*GroES H96A**	F: 5-GGCTCTTGTTGTGCTACGGATAGTCAT-3
	R: 5-TGAGCCCACAATACCTAGAATGTCTTCT-3
***Hp*GroES H100A**	F: 5-TCATACGGATAGTGCTGACCATAAACATG-3
	R: 5-CAACAAGAGCCTGAGCCCACAATAC-3
***Hp*GroES H102A**	F: 5-GATAGTCATGACGCTAAACATGCTAAAG-3
	R: 5-CGTATGACAACAAGAGCCTGAGC-3
***Hp*GroES H104A**	F: 5-CATGACCATAAAGCTGCTAAAGAGCAT-3
	R: 5-ACTATCCGTATGACAACAAGAGCCTG-3
***Hp*GroES H108A**	F: 5-ATGCTAAAGAGGCTGAAGCTTGCT-3
	R: 5-GTTTATGGTCATGACTATCCGTATGACAAC-3
***Hp*GroES H113A**	F: 5-AGCTTGCTGTGCTGATCACAAAAA-3
	R: 5-TCATGCTCTTTAGCATGTTTATGGTCAT-3
***Hp*GroES H115A**	F: 5-GCTGTCATGATGCCAAAAAACACTA-3
	R: 5-AAGCTTCATGCTCTTTAGCATGTTTA-3
***Hp*GroES H118A**	F: 5-GATCACAAAAAAGCCTAAGGTACCCC-3
	R: 5-ATGACAGCAAGCTTCATGCTCTTTAG-3
***Hp*GroES H100A/H102A**	F: 5-GATAGTGCTGACGCTAAACATGCTAAAG-3
	R: 5-ATGTTTAGCGTCAGCACTATCCGTATG-3
***Hp*GroES H100A/H108A**	F: 5-ATGCTAAAGAGGCTGAAGCTTGCT-3
	R: 5-AGCTTCAGCCTCTTTAGCATGTTTATGG-3
***Hp*GroES H102A/H108A**	F: 5-ATGCTAAAGAGGCTGAAGCTTGCT-3
	R: 5-AGCTTCAGCCTCTTTAGCATGTTTAGC-3
***Hp*GroES H113A/H118A**	F: 5-GATCACAAAAAAGCCTAAGGTACCCC-3
	R: 5-ACCTTAGGCTTTTTTGTGATCAGCAC-3

Mutation sites are underlined

F, forward; R, reverse.

Supplementary Table SII. The percentage of secondary structure content of WT and histidine mutants calculated by the CDSSTR program

Sample	Secondary structure %					
	**α Helix**	**3/10 Helix**	**β Sheet**	**β Turns**	**poly(Pro)II**	**Unordered**
**WT**	36.3	14.6	1.4	12.6	18.3	16.6
**H100A**	34.4	14.7	1.2	13.1	18.8	16.8
**H102A**	31.7	12.4	4.2	11.4	16.9	22.6
**H108A**	34.7	14.0	2.5	9.3	18.0	22.2
**H113A**	31.3	14.8	2.0	12.2	19.3	21.1
**H118A**	23.2	14.2	7.5	10.5	19.2	25.9

Reference

1 Zhang, Y. I-TASSER server for protein 3D structure prediction. *BMC Bioinformatics* **9**, 40 (2008).

2 Trott, O. & Olson, A. J. AutoDock Vina: Improving the speed and accuracy of docking with a new scoring function, efficient optimization, and multithreading. *Journal of Computational Chemistry* **31**, 455-461, doi:10.1002/jcc.21334 (2010).

3 Ryckaert, J.-P., Ciccotti, G. & Berendsen, H. J. C. Numerical integration of the cartesian equations of motion of a system with constraints: molecular dynamics of n-alkanes. *Journal of Computational Physics* **23**, 327-341, doi:http://dx.doi.org/10.1016/0021-9991(77)90098-5 (1977).

4 Darden, T., York, D. & Pedersen, L. Particle mesh Ewald: An N [center-dot] log(N) method for Ewald sums in large systems. *The Journal of Chemical Physics* **98**, 10089-10092 (1993).

5 Sindhikara, D. J., Roitberg, A. E. & Merz, K. M. Apo and Nickel-Bound Forms of the Pyrococcus horikoshii Species of the Metalloregulatory Protein: NikR Characterized by Molecular Dynamics Simulations. *Biochemistry* **48**, 12024-12033, doi:10.1021/bi9013352 (2009).
